# Two steps forward, one step back: What research progressions have been made to support the advancement of health and performance in pregnant international and world-class sportswomen since 2016? A scoping review

**DOI:** 10.1177/17455057251368289

**Published:** 2025-08-25

**Authors:** Kirsty J. Elliott-Sale, Jodie G. Dakic, Marlize De Vivo, Grainne M. Donnelly, Deirdre McGhee, Jane Thornton, Kathleen Stroia, Christopher Kronk, Melanie Hayman

**Affiliations:** 1 Department of Sport and Exercise Science, Institute of Sport, Manchester Metropolitan University, UK; 2 Hologic WTA (Women’s Tennis Association) Women’s Health Taskforce, St. Petersburg, FL, USA; 3 Department of Physiotherapy, Monash University, Frankston, VIC, Australia; 4 Advanced Wellbeing Research Centre, Sheffield Hallam University, UK; 5 Cardiff School of Sport and Health Sciences, Cardiff Metropolitan University, UK; 6 Breast Research Australia, University of Wollongong, NSW, Australia; 7 Health, Medicine and Science Department, International Olympic Committee, Lausanne, Switzerland; 8 Women’s Tennis Association, St. Petersburg, FL, USA; 9 School of Health, Medical and Applied Sciences, Appleton Institute, Central Queensland University, Rockhampton, QLD, Australia

**Keywords:** elite sportswomen, pregnancy

## Abstract

**Background:**

With more elite sportswomen incorporating pregnancy into their athletic careers, it is imperative that they are supported with evidence informed guidelines for healthy, safe pregnancies.

**Objectives:**

To (i) provide a rapid review, which mapped fields of study relevant to what is known about the health and performance-related considerations for pregnant elite sportswomen, and (ii) quantify the overall state of the art since the 2016/2017 International Olympic Committee publications on exercise and pregnancy in recreational and elite athletes.

**Eligibility Criteria:**

Original, empirical, peer-reviewed, English-language studies reporting on research conducted with or related to healthy, pregnant elite (international and world-class) sportswomen aged ⩾18 and ⩽40 years were eligible for this review. In addition, all aspects and/or metrics of health and sports performance were considered, and both quantitative and qualitative research designs were included.

**Sources of Evidence:**

PubMed, SPORTDiscus and Web of Science were systematically searched. Reviews, book chapters and grey literature were excluded. Reference lists of eligible studies were also searched to identify additional studies of relevance.

**Charting Methods:**

Scoping review with expert consultation exercise. Consideration was given to basic numerical analysis of the extent, distribution, and nature of the studies included in the review. Five key stakeholders, including physiotherapists, physicians, applied practitioners and researchers, with expertise in women’s and pelvic health, pregnancy and postpartum, breast biomechanics and rehabilitation and training in national and international-level sport took part in an expert consultation process.

**Results:**

Eight studies were identified as part of the literature review, and more than 30 topics were highlighted through consultation as areas of interest and further study related to the health and performance of elite pregnant sportswomen.

**Conclusion:**

In the last decade, an insufficient number of studies have been conducted, related to pregnant international and world-class sportswomen, meaning that the state of the art on this topic for this specific population has not changed to a noticeable degree. Experts in this area still have a plethora of unanswered research questions, such that it is still impossible to take a fully research informed approach to supporting pregnant elite sportswomen.

## Introduction

Pregnancy causes a myriad of anatomical, physiological and biomechanical adaptations that have potential implications for athlete health and performance.^1^ As such, elite athletes have generally waited until the end of their sporting careers to become pregnant.^2,3^ Today, more elite athletes are incorporating motherhood into their athletic journey.^4^ Careful consideration of timing (e.g. phase of Olympiad), funding (e.g. nature of contract), engagement (e.g. time away from training and competition) and resources (e.g. equipment and personnel) is required alongside the notable physical changes.^3^ In addition, athletes require credible, evidence-based or at least evidence-informed, guidelines to help direct their pregnancies towards a healthy, favourable conclusion and to expedite their safe return to training and performance postpartum.^3^

In 2015, the International Olympic Committee (IOC) convened an expert panel to: (i) describe the common health-related conditions that may affect strenuous training and competition during and following pregnancy; (ii) produce pregnancy and postpartum-related recommendations for training for both recreational and elite athletes and (iii) identify noteworthy omissions in the current evidence base that undermine current guidelines. This meeting resulted in a five-part series of systematic reviews,^5–9^ which covered the effects of exercise on pre-conception, gestation and postpartum, as well as providing recommendations for athletes, practitioners and future research. With specific reference to future research, the expert panel proposed a large number of research questions (approximately 40 in total) related to pregnancy, labour and birth and the postpartum period, thus highlighting the lack of evidence available at that time and the urgent need for further investigations. Their pregnancy-specific questions were related to medical conditions (e.g. ‘Is heavy work or strenuous exercise associated with miscarriage?’), physiological and anatomical changes (e.g. ‘What is the prevalence of, and what are the risk factors for, diastasis recti abdominis during pregnancy in elite athletes?’), exercise testing (e.g. ‘Which testing protocols are safe and appropriate for use in pregnant elite athletes?’), athletic training (e.g. ‘What is the effect of flexibility training on range of motion during pregnancy?’) and exercise interventions (e.g. ‘Can abdominal exercise during pregnancy prevent and/or treat diastasis recti abdominis?’). These questions, reflecting omissions in the current evidence-base, provided a timely research agenda for future studies in this area.

It is worth noting that the studies included in the IOC’s publications reflected the research and applied landscape faced by sportswomen at the Rio 2016 Olympic Games. Female participation rates at the Games have increased – from 45% of all athletes at the Rio 2016 Games to 50% at the Paris 2024 Games – and the number of Olympians having babies within their career has increased. It is unknown whether research rates and knowledge generation have developed in line with these statistics? As such, the purpose of this scoping review was to identify the research outputs in the ensuing years since the expert panel met and published their pregnancy-specific findings and recommendations for female athletes and document when their proposed pregnancy-specific questions were addressed. It is important to note that whilst the IOC publications^5–9^ included both recreational and elite female athletes this scoping review was focussed on data related to elite athletes only. Likewise, whilst the IOC published recommendation on postpartum return to sport this review focuses on recommendations specific to pregnancy. Therefore, our research question was: *what research advancements have been made in relation to the health and performance-related considerations for pregnant elite sportswomen between the 2016 and 2024 Olympic Games?* Consequently, the aims of this review were to (i) examine the extent, range, and nature of research activity related to the health and performance considerations of pregnant elite sportswomen in the last 8 years and (ii) identify research gaps in the literature. The objectives were to: (i) provide a rapid review, which mapped fields of study relevant to the aim; and (ii) quantify the overall state of the art (i.e. inclusions and omissions). In achieving these aims and objectives, we can also speak to a more fundamental question related to participation rates (i.e. the number of women participating at the elite level) versus knowledge acquisition (i.e. the number of research publications addressing issues related to elite sportswomen).

## Methods

### Protocol

This scoping review conformed to the Preferred Reporting Items for Systematic Reviews and Meta-Analyses Extension for Scoping Reviews methodological guidelines.^10^ The checklist can be found in Supplemental Material. It was also conducted in accordance with the five-stage framework for scoping reviews (i.e. a literature search) and included an optional consultation exercise.^11^ This review protocol was not registered or published.

### Eligibility criteria and search strategy for literature search

To be considered eligible, the research had to be conducted with or related to pregnant elite sportswomen aged ⩾18 and ⩽40 years thus representing adult premenopausal women (i.e. prime reproductive years). Participants’ training status (i.e. elite) was classified using the criteria outlined by McKay et al.^12^ For the purposes of this review, participants had to be Tier 4 or 5; reflecting international (~0.0025% of the global population) to world-class (<0.00006% of the global population) sportswomen. Participants were either pregnant at the time of study or had already had their babies and as such were providing their data retrospectively. Participants had to be defined as healthy (i.e. not having any medical condition or taking any medication known to affect any of the outcome measures) and had to provide details on stage of gestation. Singleton and multiple pregnancies and athletes of all gravidity and parity were considered. All aspects and/or metrics of health and sports performance were considered. Both quantitative and qualitative research designs were included. For the purpose of this scoping review, the terms ‘athlete’ and ‘sportswomen’ have been used interchangeably.

We searched PubMed, SPORTDiscus and Web of Science for studies published between January 2015 and December 2023. The lower date limit was applied as this was when athlete-facing information^9^ were last published. In addition, Barakat et al.^13^ published a narrative review, around this time, on exercise during pregnancy (not in elite sportswomen), asking: ‘what do we know?’. As such, the current scoping review provides an update on the research published since then (i.e. excluding the IOC recommendations and Barakat review). The upper date limit was applied as the information provided by the review was needed to inform time-critical decision-making regarding future research priorities. We only searched for English-language studies. Foreign language material was excluded because of the cost and time involved in translating material. Original, empirical and peer-reviewed studies were included, whilst reviews, book chapters and grey literature were excluded, in order to showcase new data produced within the given timeframe. Reference lists of eligible studies were also searched to identify additional studies of relevance. We acknowledge that, whilst these limits were adopted for practical reasons, potentially relevant articles could have been missed.

Searches were conducted with the following terms:

Web of science– ALL=((‘pregnancy’ OR ‘gestation’ OR ‘pregnant women’ OR ‘pregnant’ OR ‘antenatal’ OR ‘trimester’ OR ‘prenatal’ OR ‘perinatal’) AND (‘elite athlete’ OR ‘elite’ OR ‘athlete’ OR ‘Olympic’ OR ‘world-class’) AND (‘athletic performance’ OR ‘muscular strength’ OR ‘strength training’ OR ‘muscular force’ OR ‘power’ OR ‘anaerobic’ OR ‘anaerobic power’ OR ‘anaerobic capacity’ OR ‘aerobic’ OR ‘aerobic performance’ OR ‘endurance’ OR ‘endurance capacity’ OR ‘endurance performance’ OR ‘fatigue’ OR ‘recovery’ OR ‘health’ OR ‘pelvic’ OR ‘pelvic health’ OR ‘breast’ OR ‘maternal health’))
PubMed – ((pregnancy) OR (gestation) OR (pregnant women) OR (pregnant) OR (antenatal) OR (trimester) OR (prenatal) OR (perinatal)) AND ((elite athlete) OR (elite) OR (athlete) OR (Olympic) OR (world-class)) AND ((athletic performance) OR (muscular strength) OR (strength training) OR (muscular force) OR (power) OR (anaerobic) OR (anaerobic power) OR (anaerobic capacity) OR (aerobic) OR (aerobic performance) OR (endurance) OR (endurance capacity) OR (endurance performance) OR (fatigue) OR (recovery) OR (health) OR (pelvic) OR (pelvic health) OR (breast) OR (maternal health))
SPORTDiscus – (‘pregnancy’ OR ‘gestation’ OR ‘pregnant women’ OR ‘pregnant’ OR ‘antenatal’ OR ‘trimester’ OR ‘prenatal’ OR ‘perinatal’) AND (‘elite athlete’ OR ‘elite’ OR ‘athlete’ OR ‘Olympic’ OR ‘world-class’) AND (‘athletic performance’ OR ‘muscular strength’ OR ‘strength training’ OR ‘muscular force’ OR ‘power’ OR ‘anaerobic’ OR ‘anaerobic power’ OR ‘anaerobic capacity’ OR ‘aerobic’ OR ‘aerobic performance’ OR ‘endurance’ OR ‘endurance capacity’ OR ‘endurance performance’ OR ‘fatigue’ OR ‘recovery’ OR ‘health’ OR ‘pelvic’ OR ‘pelvic health’ OR ‘breast’ OR ‘maternal health’)

### Selection of sources of evidence

Two reviewers (KES/CS) independently applied the inclusion and exclusion criteria to all identified citations. Following the title and abstract screening process, full-text articles were obtained for all relevant studies and for studies where the relevance was unclear from the initial vetting stage. Both reviewers assessed the eligibility of citations by reading the full articles. Conflicts were resolved through discussion, until a majority consensus was reached. All conflicts were resolved between reviewers KES and CS. As such, a third-party reviewer was not required to reach a majority-based consensus. Cohen’s kappa (κ) was used to assess inter-rater agreement of articles for full-text review: <0 indicates no agreement; 0.01–0.20 as none to slight agreement; 0.21–0.40 as fair agreement; 0.41–0.60 as moderate agreement; 0.61–0.80 as substantial agreement and 0.80–1.00 as almost perfect agreement.^14^

### Data charting process and data items

Using a customised Microsoft Excel spreadsheet, we charted and interpreted the following qualitative data: author(s), year of publication, study location; study design; study population (i.e. training status/tier, sport, stage of gestation, gravidity and parity); aims of the study; methodology, outcome measures; important results/recommendations and noteworthy omissions and limitations. All data were extracted by one reviewer and cross-referenced by a second reviewer. Consideration was given to basic numerical analysis of the extent, distribution and nature of the studies included in the review.

### Consultation exercise

The consultation exercise identified current issues within the field that remain under-researched. Key stakeholders with expert knowledge in the fields of breast biomechanics, rehabilitation and training, women’s health including pelvic floor, pregnancy and postpartum health and sports medicine were consulted with. Stakeholders included representatives from the following professions: physiotherapy, sports medicine physicians, sports and exercise scientists and researchers. The experts were identified based on their experience in practising and/or researching in national and international level sport. The stakeholders were from multiple geographies. This group provided insights about issues relating to the health and performance-related considerations of pregnant elite sportswomen. Their input was sought and provided by numerous email exchanges; in which they were required to complete the questions displayed in Table 1.

**Table 1.** Input provided by key stakeholders as part of the expert consultation exercise.

Qualification[s] and current role[s]
What is your area of expertise/specialism/ experience?
Have you researched/worked with/supported elite sportswomen? Yes/No
Have you researched/worked with/supported pregnant elite sportswomen? Yes/No
In your opinion, what are the health-related considerations for pregnant elite sports women?
In your opinion, what are the performance-related considerations for pregnant elite sports women?
In your opinion, which health-related issues, with regards to pregnant elite sportswomen, are under-researched?
In your opinion, which health-related issues, with regards to pregnant elite sportswomen, are well-researched? Please comment on the quantity and quality of this research.
In your opinion, which performance-related issues, with regards to pregnant elite sportswomen, are under-researched?
In your opinion, which performance-related issues, with regards to pregnant elite sportswomen, are well-researched? Please comment on the quantity and quality of this research.

## Results

### Selection of sources of evidence

**Figure 1.**
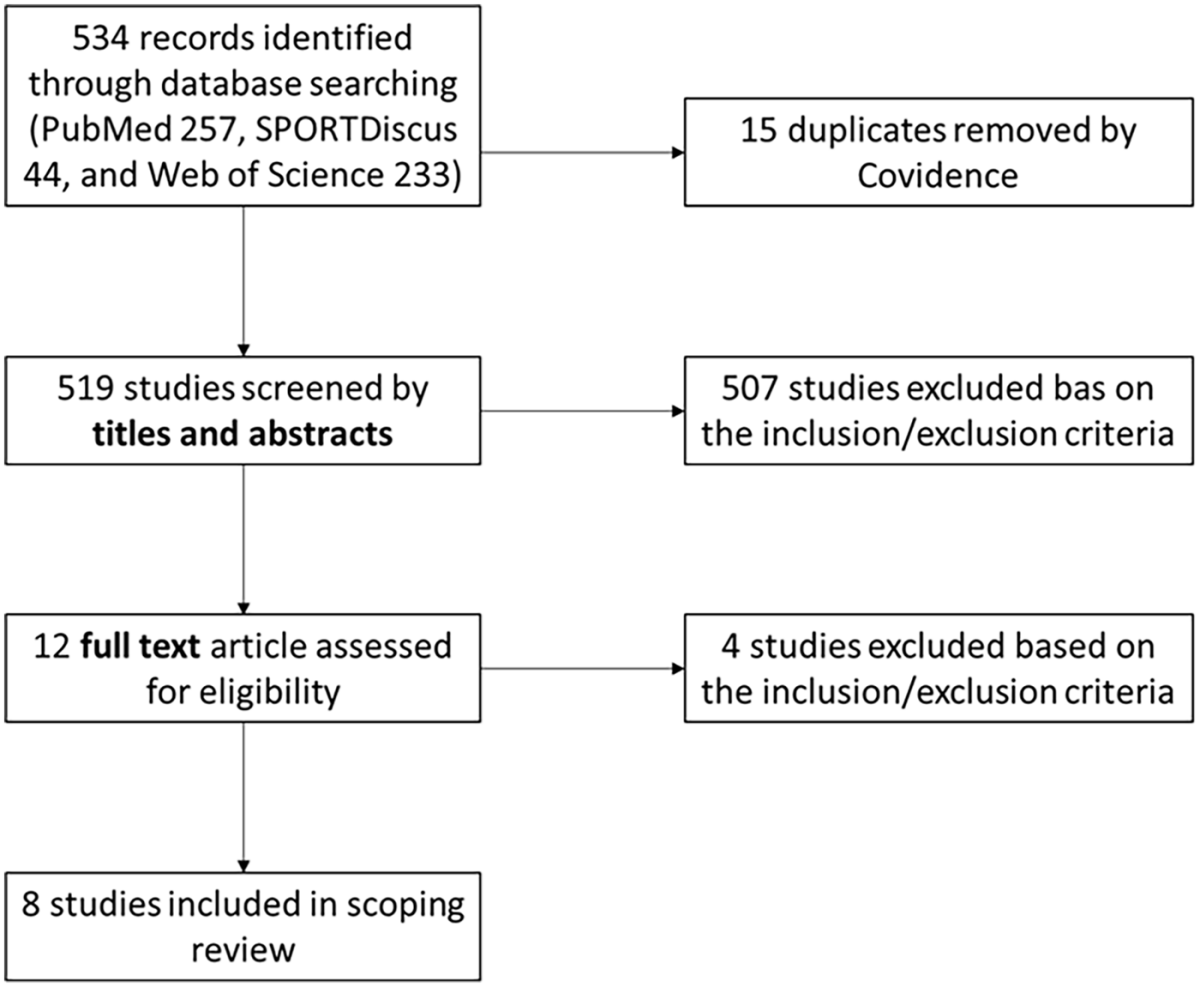
PRISMA flowchart. PRISMA: Preferred Reporting Items for Systematic Reviews and Meta-Analyses.

Cohen’s κ was 0.9 for inter-rater agreement of articles for full-text review, which indicates ‘almost perfect agreement’ between reviewers. The percentage of agreement between reviewers was 99.8%. A third reviewer was not needed for conflict resolution. Figure 1 shows the search and selection process.

### Characteristics of sources of evidence

#### Extent (volume and range) of research activity

Between 2015 and 2023, eight studies were published that were deemed eligible for this scoping review: Darroch et al.^15^; Davenport et al.^16,17^; Massey and Whitehead^18^; Sundgot-Borgen et al.^19^; Solli and Sanbakk^20^ and Martinez-Pascual et al.^21,22^

#### Distribution (frequency and type) of research activity

One article was published per year between 2016 and 2019, and two articles were published per year in 2022 and 2023. Studies employed qualitative,^16–18,21,22^, quantitative^15^ and mixed methods approaches,^19,20^ including retrospective questionnaires (*n* = 2), interviews (*n* = 7), published online data (*n* = 1), physical testing (*n* = 1) and notes, letters and diaries (*n* = 3).

#### Nature (characteristics) of research activity

##### Sports

Participants (athletes, coaches, sports medicine physicians, and physiotherapists) were described in a number of ways making it difficult to specify every sport included within this scoping review. For example, some participants were (i) described using umbrella terms (e.g. endurance sports, ball-sports, aesthetic sports, weight class sports, technical sports, team sports and individual sports); (ii) listed by sport (e.g. triathlon, archery, javelin, short distance running, high jump, long distance running including marathon, handball, rugby, tennis, canoeing, basketball, taekwondo, judo, cross-country skiing); (iii) identified by their Olympic event (e.g. Olympic discus thrower and Paralympic sprinter) and (iv) categorised by their competitive level (e.g. athletes who have trained and/or competed at the highest level of their [not stated] sport). In line with the inclusion criteria, all studies included Tier 4 and 5 athletes, either directly as participants or indirectly in the form of staff who supported this level of athlete. Table 2 shows the characteristics of each included study.

**Table 2.** Nature (characteristics) of research activity.

Author	Study design/data collection method	Participants			
		Role	Sport	Residential country	Gestation/parity
Darroch et al.^15^	Quantitative; retrospective self-report online questionnaire	Female athletes (*n* = 42)	Elite/world class runners (middle and endurance >1500 m)	Global (Australia, Canada, Ireland, Lithuania, Monaco, United Kingdom, and the United States of America.)	Parity unreported Reported on first pregnancy only
Davenport et al.^16^	Qualitative; semi structured interviews	Female athletes (*n* = 20)	Varied Olympic sports	U.S. and Europe	Pregnant in the last 5 years
Davenport et al.^17^	Qualitative; semi structured interviews	Coaches (*n* = 5), three sports medicine physicians (*n* = 3) and physiotherapists (*n* = 8) – working with pregnant/post-partum athletes in the last 5 years Gender: Women (*n* = 13), men (*n* = 3)	Broad range of sports	Primarily U.S.	Not reported
Massey and Whitehead^18^	Qualitative; semi structured interviews	Female athlete	Olympic discus thrower (*n* = 1) Paralympic sprinter (*n* = 1)	Not reported	Pregnant with first child
Martinez-Pascual et al.^21^	Qualitative; unstructured and semi-structured interviews	Female athletes (*n* = 20)	Varied sports (triathlon, javelin, middle distance running, high-jump, marathon, handball, basketball, rugby, tennis, canoeing, taekwondo and judo)	Spanish	Had been pregnant and returned to professional sport for at least 1 year. Parity between one and four births.
Martinez-Pascual et al.^22^	Qualitative; unstructured and semi-structured interviews	Female athletes (*n* = 20)	Varied sports (triathlon, javelin, middle distance running, high-jump, marathon, handball, basketball, rugby, tennis, canoeing, taekwondo and judo)	Spanish	Had been pregnant and returned to professional sport for at least 1 year. Parity between one and four births.
Sundgot-Borgen et al.^19^	Quantitative; purposed designed questionnaire	Elite female athletes (*n* = 34) and active female controls (*n* = 34)	Varied sports	Norwegian	Pregnant and gave birth in previous 5 years. Parity: Pregnant with first child (*n* = 28 athletes, 30 controls); multiparous (*n* = 6 athletes and 4 controls)
Solli and Sanbakk^20^	Quantitative, case-study	Female athlete (*n* = 1)	Cross-country skiing	Norway	First birth

##### Location

The studies were conducted (i.e. based on where ethical approval was granted and/or where the majority of the research team was based) in Canada (*n* = 3), the United Kingdom (*n* = 1), Norway (*n* = 2) and Spain (*n* = 2), although it should be noted that, as some studies recruited participants from social media, the participants were from a much wider [not stated] global pool.

##### Gestation

The majority of studies recruited participants who were either pregnant (*n* = 1) or less than 5 years from parturition (*n* = 4) or associated with such athletes; one study recruited participants who were within the first 2 months of motherhood, one study (two publications) recruited participants who had been pregnant during their sporting professional career and one case study was based on an elite female athlete from conception to 61 weeks postpartum. All studies examined pregnancy, either in real-time or retrospectively. Details related to gravidity, parity and singleton versus multiple pregnancy were scarcely reported; four studies specifically mentioned parity (data collected related to ‘first’ child only – Dannoch et al.,^15^ Massey and Whitehead^18^; Solli and Sandbakk^20^ and data collected related to ‘last’ child only – Sundgot-Borgen et al.^19^) and one study mentioned singleton pregnancy.^20^

##### Outcomes

Five studies used an approach (i.e. qualitative, self-reported lived experiences) capable of identifying a potential mixture of health and performance-related considerations.^16–18,21,22^ Two studies focussed primarily on performance-related outcomes (e.g. pregnancy-related training volume, type, modality and intensity, as well as pre-pregnancy and postpartum competitive performance^15,20^ and one study incorporated both health and performance-related outcome measures.^19^ With the exception of a small number of objective outcome measures from two studies (i.e. changes in physiological and anthropometric parameters^20^ and competitive performance scores^15^), all other outcomes were self-reported [mostly retrospectively] and as such can be considered as subjective outcome measures.

##### Findings

Five studies^16–18,21,22^ reported numerous themes relevant to the research question including: (i) lack of female athlete reproductive research; (ii) need to develop evidence-based progression for sport participation in pregnancy and postpartum; (iii) essential supports and changes required for pregnant athletes; (iv) training pregnant athletic bodies; (v) safety concerns; (vi) a new body; (vii) body control; (viii) to feel their bodies and communicate with them; (ix) fears and doubts; (x) justifying physical exercise; (xi) physical identity and (xii) dual identity. It is worth noting that although the search strategy was focussed on pregnant and not postpartum elite female athletes, several of the articles included in this review contained some postpartum data. Only the pregnancy-related data are discussed here.

Two studies^15,20^ reported quantitative training and competition-related data: (i) running volume decreased significantly from the first trimester to the third trimester; (ii) during the first and second trimesters, the average training volume was 80%–85% of pre-pregnancy values, but then progressively decreased to 50% during the third trimester where training was gradually reduced throughout; (iii) whilst light and moderate-intensity exercise was performed throughout pregnancy, no high-intensity exercise was performed after gestational week 5 and strength training was progressively modified and (iv) by reducing the overall training load, slower progression and utilisation of alternative exercise modes, the participant had a successful training development.

Lastly, Sundgot-Borgen et al.^19^ demonstrated no group differences in fertility problems, pregnancy loss, preterm birth or low birth weight between Norwegian elite female athletes from a broad range of sports (*n* = 34) and active controls (*n* = 34). Both groups decreased training volume in all trimesters and the first two postpartum periods compared with pre-pregnancy, and more athletes than active controls returned to sport/exercise at week 0–6 postpartum. There were no group differences in complications during pregnancy and delivery, but athletes reported fewer common complaints. Athletes were not satisfied with advice related to strength training and nutrition during pregnancy.

##### Omissions and limitations

All studies reported limitations and subsequent omissions. The majority of issues related to diversity, for example: (i) gender bias in a study exploring professional practitioners lived experience working with pregnant athletes, a majority of participants identified as women with a personal experience of pregnancy, thereby limiting data on the experiences of male practitioners and/or those without experience of personal pregnancy; (ii) volunteer bias whereby those with very positive or neutral experiences may have chosen not to participate; (iii) location bias wherein low-income or middle-income countries were largely excluded; (iv) sport discipline bias therefore skewing sport-specific effects; (v) recall bias thereby increasing the risk of under/over-reporting and (vi) desirability bias and therefore under-reported complaints or complications and/or under/over-reported training volume in the different periods. Lastly, as the majority of studies were either exclusively qualitative or mixed methods with a qualitative component, it should be noted that these types of studies make it difficult for researchers to generalise these data.

### Gaps in current literature

In 2017, the IOC expert panel^8^ posed 21 priority pregnancy-related research questions, based on 3 systematic reviews,^5–7^ which related to fertility, medical conditions, physiological and anatomical changes, exercise testing, athletic training, exercise interventions and labour and birth. The data collected since then loosely addresses, both directly and indirectly, some of these research priorities. We assert, however, that none of the topics highlighted by the IOC expert panel^8^ have been completely resolved, meaning that their questions remain either unanswered or unexplored. Moreover, the quality of data identified as part of this scoping review has not been assessed meaning that we cannot comment on our confidence in the latest findings. Lastly, the majority of studies included in this scoping review were qualitative in nature, highlighting a dearth of quantitative data related to the unresolved gaps in current literature.

### Consultation exercise

**Figure 2.**
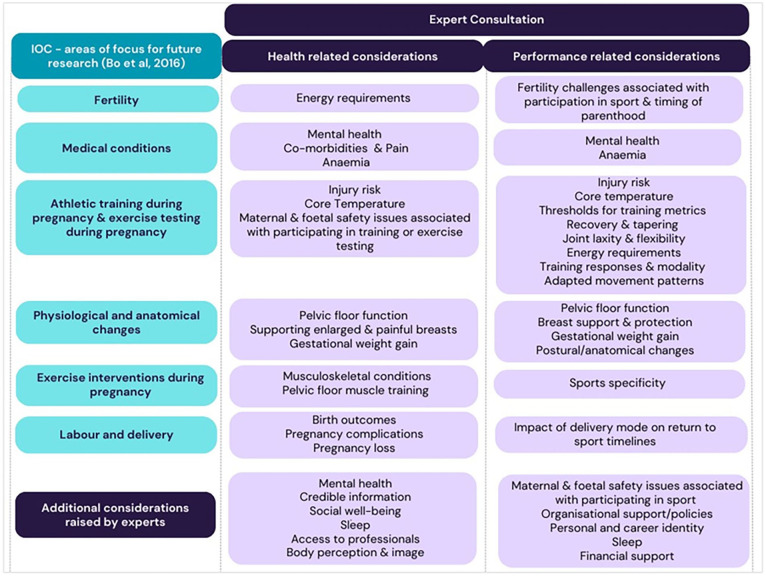
Unexplored research topics related to pregnancy in elite sportswomen identified through the expert consultation exercise as lacking evidence, mapped against topics identified in the IOC review. IOC: International Olympic Committee.

When asked ‘What are the health/performance related considerations for pregnant elite sports women?’ more than 30 topics were identified. In response to ‘Which health/performance related issues, with regards to pregnant elite sportswomen, are under-researched?’ there was universal agreement that all of the listed considerations were under-researched. Eight topics were highlighted as being commonly researched (low back pain, benefits of being active, injury rates, motherhood identity, case studies, postpartum peak performance and postpartum recovery), although all of these points were described as still needing further high-quality evidence. Figure 2 summarises the findings, listed in no particular order, of the consultation exercise. The topics identified in Figure 2 represent a multiplicity of research questions.

## Discussion

### Summary of the evidence

In the last 7 years, since the IOC published their reviews,^5–9^ just eight studies^15–22^ have been published, which relate to health and performance considerations for pregnant elite (defined as international, world-class) sportswomen despite the considerable growth in women’s sport and the number of elite female athletes experiencing motherhood during their athletic careers. Whilst offering significant insight to the field, most of these studies used retrospective, self-reported (i.e. subjective) outcome measures,^16–19,^21,22^^ with just two studies reporting objective outcome measures.^15,20^ All studies described numerous and significant limitations and omissions. The bulk of the research questions posed by the IOC in 2018 have still not been addressed and remain unanswered, noting that the IOC posed questions related to recreational and elite (national level and above), whereas the present scoping review was focussed on international and world-class athletes only. Similar to the literature review, the expert consultation detailed a multitude of unexplored topics related to pregnancy in elite sportswomen, with each topic representing many outstanding research questions (i.e. 40 topics each with 3–5 specific research questions, resulting in upwards of 150 required studies). These data suggest that little has changed in the last decade in this research area and that considerably more research attention is needed, which focusses on the health and performance of pregnant elite sportswomen. None of the studies, for example, included recommendations to manage breast-related issues associated with increased breast size during pregnancy despite it being a modifiable barrier to performance in female athletes.^23^ Larger breast size is known to negatively affect performance and participation in sports in female athletes and active women through excessive breast movement and associated breast pain, bra discomfort, chafing to the skin underlying brassieres, increased thoracic kyphosis and increased risk of breast injuries.^23,24^

In the absence of a significant body of new data, pregnant elite athletes and their entourage should still be encouraged to familiarise themselves with the IOC recommendations for elite athletes experiencing healthy pregnancies^9^ whilst also recognising progress in the recommendations and research involving the general pregnant population. In this context, the World Health Organization^25^ advises that when considering athletic competition or exercising significantly above the recommended guidelines for being active during pregnancy, women should seek supervision from a specialist healthcare provider. In addition to continuous clinical monitoring, screening tools such as the Get Active Questionnaire for Pregnancy,^26^ should be administered regularly to identify potential contraindications or underlying medical conditions (see Meah et al.^27^). When such conditions are detected, adjustments to the pregnant athlete’s exercise regimen are advised, including modifications, reductions, or, in certain cases, the cessation of moderate to vigorous intensity activities. Submaximal exercise testing protocols are recommended for healthy pregnant athletes^28^ and where exercise is not contraindicated, the principles of exercise programming should be considered alongside the significant anatomical and physiological changes that occur during pregnancy. Consideration should be given to the following topics: hyperthermia, prolonged activities in the supine position, altitude, nutrition, exercise intensity, strength training, adequate breast support for increased breast size^9,23^ and appropriate exercise loading for individual pelvic floor function. In addition, advice provided by the IOC on common complaints and diseases during pregnancy, such as nausea, fatigue, pre-eclampsia and birth outcomes, such as pre-term birth, prolonged labour and caesarean sections should be disseminated to pregnant elite athletes or those who may be planning. These recommendations should be viewed alongside the findings from the studies identified herein,^15–22^ which chart the lived experiences, training and competitive data, and pregnancy and birth outcomes of pregnant elite sportswomen.

### Strength and limitations

As with all review articles, the methodological approach employed might have resulted in the potential omission of relevant data; for example:

1. By a priori design, this scoping review did not appraise the quality of evidence in the identified studies; instead, it focussed on the breadth (i.e. finding all) rather than the depth (i.e. describing and analysing the findings) of current literature. As such, it does not address the relative weight of evidence on the health and performance-related considerations for pregnant elite sportswomen, instead we have provided a descriptive account of available literature and evidence gaps.
2. The strict eligibility criteria for the search and selection process, in particular the use of both upper and lower date restrictions rather than an open timeframe, might have limited the identification of relevant material as opposed to a more expansive remit.
3. Whilst every attempt was taken to identify key stakeholders to participate in the expert consultation, the somewhat subjective selection process might have biased these findings.

Using a rapid review approach, we have provided and undertaken a rigorous and transparent method for mapping this area of research, meaning that we have been able to illustrate the field of interest in terms of the extent, distribution and nature of the current literature. In addition, this approach has made it possible to identify gaps in the existing evidence base. Moreover, the framework employed in this scoping review included a role for key stakeholders to provide additional insights regarding issues related to the health and performance-related considerations of pregnant elite sportswomen, allowing us to extend the scope of the review from solely published literature to current issues within the field of investigation. By undertaking these processes and presenting the results in this format, researchers and practitioners are better placed to direct future research in this area.

### Future research directions

In order to provide evidence-based recommendations to guide elite sportswomen through pregnancy, maximising their health well-being and exercise capacity, extensive future studies are needed. In particular with a focus on:

- Quantitative, longitudinal and observational cohort studies with surveillance of health, physiological and training outcomes to allow development of future clinical guidelines and exercise/training frameworks.
- Studies focussed on elite sportswomen due to the specific demands required by this population and lack of focussed research in this cohort
- Rigorous reporting of research outcome measures and interventions to enhance reproducibility of research across sports globally due to the small sample sizes available in elite populations.

## Conclusions

The lack of research attention and evidence makes it difficult for international and world-class sportswomen and their practitioners to employ an evidence-informed approach to managing pregnancy during their career. To produce up-to-date and fit-for-purpose guidelines for the growing number of international and world-class sportswomen incorporating pregnancy into their athletic careers, immediate and widescale research is needed on the health and performance considerations for these women.

## Supplemental Material

sj-docx-1-whe-10.1177_17455057251368289 – Supplemental material for Two steps forward, one step back: What research progressions have been made to support the advancement of health and performance in pregnant international and world-class sportswomen since 2016? A scoping review Supplemental material, sj-docx-1-whe-10.1177_17455057251368289 for Two steps forward, one step back: What research progressions have been made to support the advancement of health and performance in pregnant international and world-class sportswomen since 2016? A scoping review by Kirsty J. Elliott-Sale, Jodie G. Dakic, Marlize De Vivo, Grainne M. Donnelly, Deirdre McGhee, Jane Thornton, Kathleen Stroia, Christopher Kronk and Melanie Hayman in Women's Health
